# Identification and quantification of target metabolites combined with transcriptome of two rheum species focused on anthraquinone and flavonoids biosynthesis

**DOI:** 10.1038/s41598-020-77356-9

**Published:** 2020-11-20

**Authors:** Jing Liu, Liang Leng, Yan Liu, Han Gao, Wei Yang, Sha Chen, An Liu

**Affiliations:** Key Laboratory of Beijing for Identification and Safety Evaluation of Chinese Medicine, Institute of Chinese Materia Medica, China Academy of Chinese Medical Sciences, No. 16, Nanxiaojie, Dongzhimennei, Beijing, 100700 China

**Keywords:** Biochemistry, Biological techniques, Molecular biology, Plant sciences

## Abstract

*Rheum emodi* is a perennial herb and an important medicinal plant, with anthraquinones and flavonoids as its main bioactive compounds. However, there is little knowledge about the biosynthetic pathway of anthraquinones in rhubarbs. In this study, we qualitatively and quantitatively assessed 62 pharmacological metabolites in rhubarb using dynamic multiple reaction monitoring (dMRM) of triple-quadrupole mass spectrometry (QqQ-MS), including 21 anthraquinones, 17 flavonoids, 6 stilbenes, 12 gallate esters, 3 tannins, and 3 others. Besides, the metabolomics results showed significant differences among all the 60 metabolites, except for gallic acid and piceatannol-*O*-β-glucoside. The combined transcriptome data of *R. palmatum L*. (RPL) and *R. officinale Baill.* (ROB) showed that 21,691 unigenes were annotated in the metabolic pathways. Taken together, 17 differentially expressed genes (DEGs) were associated with the anthraquinone biosynthetic pathway. Additionally, a significant correlation between anthraquinone peak intensity and DEG expression level existed, validating that DEGs contribute to the anthraquinone biosynthetic pathway. RT-qPCR results showed that the cluster-14354.38156 gene may catalyze the O-methylation of emodin to produce physcion. This study provides a useful resource for further studies on secondary metabolism in rhubarb and the combination analysis of transcriptome and metabolome, which can help with the discovery of enzyme genes involved in metabolite biosynthesis.

## Introduction

*Rheum emodi*, also called “Dahuang”, is a plant belonging to the species of the family Polygonaceae, and is commonly used as a medicinal plant due to its rich content of diverse pharmaceutically important secondary metabolites, especially anthraquinones^1^. In ancient China and Western countries, rhubarb has mainly been used to reduce body clarity and ease excretion. China has been the major global rhubarb producer. Rhubarb has been in great demand in Europe owing to the local eating habits. Rhubarb has also been used as an effective, quick-acting, and painless cathartic^2^. The utilization of its roots and rhizomes had been frequently featured in traditional Chinese Pharmacopeia, e.g., *Shennong Bencaojing* (written between 200 and 300 AD) and *Bencao Gangmu* (written in 1593), and among the three identified species of rhubarb, two are widely used in clinical medicine, namely *R. palmatum L*. (RPL) and *R. officinale Baill.* (ROB)^3^. The main bioactive compounds in rhubarb are anthraquinones^4^ and flavonoids. However, the differences in the composition of the pharmaceutical metabolites between the two rhubarb species (RPL and ROB) remain uninvestigated.

The main anthraquinone derivative in rhubarb is emodin anthraquinone. Currently, there are approximately more than 30 anthraquinone compounds discovered in rhubarb, including emodin, aloe-emodin and physcion^5^. As to the same skeletal construction, different structural modifications endow these metabolites with different pharmacologic effects^6^. There are two known distinct and major anthraquinone biosynthetic pathways in higher plant species, including the polyketide pathway and chorismate/*o*-succinylbenzoic acid pathway, and the former leads to the biosynthesis of emodin-type anthraquinones and flavonoids^7,8^. In the polyketide pathway, acetyl coenzyme-A and malonyl coenzyme-A were taken as the initial and extension units, respectively, and the phenyl ring structure was formed by condensation and cyclization under the action of polyketide synthase, which then exhibit a characteristic substitution pattern in their aromatic rings^9^. Under the catalysis of plant-specific type III polyketide synthase, the precursor of free anthraquinone, anthrone, is generated through aldolization, enolization, oxidation and decarboxylation. Then, free anthraquinone is converted into bound anthraquinone through the action of glycosyltransferase^10,11^. Similarly, the initial synthesis of flavonoids is also influenced by polyketide synthase, with malonyl coenzyme-A as the extender unit^12–15^. Chalcone, a flavonoid precursor, is synthesized by plant-specific type III polyketide synthase through aldolization and isomerization, which is further catalyzed by chalcone isomerase to produce free flavonoids. Flavonoid glycosides are produced through the action of glycosyltransferase.

As far as we know, scholars at home and abroad mostly focus on the chemical composition and pharmacologic properties of rhubarb, and there is little progress on the molecular level^9,16–20^. The anthraquinone biosynthetic pathway has not been elucidated as clear as the flavonoid biosynthetic pathway, although biosynthesis of anthraquinones in rhubarb has been the main purpose of several previous studies^21,22^. The results from investigating the metabolic pathway of anthraquinones are also limited in the currently available databases. Further studies on the molecular level of rhubarb are meaningful and need more effort. In the post-genome era, integrated analysis of comprehensive gene expression (transcriptome) and metabolic profiling (metabolome) data has been successfully applied on plant functional genes^23,24^. Since knowledge about the biosynthetic pathway of anthraquinones in rhubarb is insufficient, the combination of technologies, such as plant metabolomics, high-throughput transcriptome sequencing, and qRT-PCR, was applied to study the anthraquinone and flavonoid pathways in our study. Additionally, owing to the lack of annotations of the anthraquinone metabolic pathway, the biosynthesis pathway of rhubarb was investigated with reference to the flavonoid metabolic pathway, a polyketide pathway. Our study may serve as a resource for future research of rhubarb, which lays a solid foundation for the molecular mechanism of anthraquinone biosynthesis in rhubarb.

## Results

### Mass conditions

The metabolic analysis of *R. officinale Baill.* (ROB) and *R. palmatum L*. (RPL) leaves were optimized by comparing the performance of several candidate elution systems, based on previous reference data^25^. The acidity of the elution system was the most important factor for the analysis. Therefore, different elution acidities with 0.1%, 0.2%, 0.5%, and 0.8% formic acid were evaluated by comparing the obtained target compounds and resolution of all metabolites. Finally, 0.2% formic acid exhibited the best performance. The optimized HPLC and electrospray ionization-QqQ-MS (ESI-QqQ-MS) conditions were as follows: the solvent system was milli-Q water containing 0.2% formic acid (eluent A) and acetonitrile (eluent B). All samples were analyzed using an Eclipse-Plus C_18_ column (2.1 mm × 50 mm, 1.8 μm) at 30 °C with a linear elution gradient protocol of 0–1.5 min, 5% B; 1.5–3 min, 5–24% B; 3–5 min, 24–25% B; 5–11 min, 25–66% B; 11–16 min, 66% B; and 16–17 min, 66–100% B; with a flow rate of 0.2 mL/min. Under these conditions, the metabolites of rhubarb leaves were completely eluted with the best target resolution (Figure S1).

### Identification of metabolites in rhubarb leaves based on ESI-Q-TOF–MS/MS

The identification of metabolites based on the ESI-Q-TOF–MS performed in both positive and negative ESI modes, were used to determine fragment ion information of metabolites. Data, including mass spectra (in PI, NI, NI-MS^2^, and PI-MS^2^), ultraviolet-vis spectra, and retention time on the C_18_ column are list in Table 1. The major 21 anthraquinones (Compound 1–21) isolated from rhubarb leaves, comprised five main types of free anthraquinone, including aloe emodin, rhein, emodin, chrysophanol, physcion, which corresponded to the characteristic fragment ions m/z 269.1, 283.1, 269.1, 253.1, 283.1 at negative mode, respectively, which were also unambiguously characterized with authentic standards, whereas the other 14 compounds were anthraquinone conjugates. Compounds 7 were identified as chrysophanol-*O*-glucoside with the molecular weight 416.1, for which a series of fragment ions m/z 253.1 for characteristic free anthraquinone-chrysophanol and the neutral losses of 162 Da, indicating that glucoside were mostly cleaved from anthrquinones has been detected from rhubarb^27^. It was observed that the two pair of isomers, including physcion and rhein, as well as emodin and aloe-emodin, can be differentiated due to their different mass spectrometry behaviors^27^. Physcion fragmentation was initiated by eliminating a methyl group, followed by the loss of CO to give m/z 240.1, whereas rhein produced the ion at m/z 239.1 by degrading the phenyl ring (–C_2_H_2_) and cleavage of the carboxyl group (–CO_2_), which was used for identification of compounds 3,11, 13,14, 15,17. Regarding emodin and aloe-emodin, the former easily eliminated CO and further lost one hydroxyl group to produce m/z 225.1, whereas the later only produced the fragment at m/z 240.1 ([M–H–CHO]^−^). Similar to free anthraquinones, anthraquinone conjugates easily produced [M–H]^−^ ions in the negative ESI source. The MS/MS fragmentations of anthraquinone glycosides were predominated by elimination of the glucosyl residue to give [aglycone–H]^−^ ions as the base peak. The corresponding aglycones were identified based on the [A–H]^−^ ions spectra, with reference to the MS fragment behaviors of free anthraquinones. The obvious difference in MS/MS spectra between free anthraquinone isomers sufficed their discrimination, and was valuable for identifying their corresponding glycosides. Based on combined data available on the mass spectra with molecular weight (PI-MS, and/or NI-MS) and characteristic fragment ions (NI-MS2, and/or PI-MS2) other anthraquinone were identified, which was been previously identified in rhubarb^28–31^.

**Table 1:** Qualitative result of metabolites in rhubarb leaves.

	No	Identification	Rt (min)	NI-MS	NI-MS^2^	PI-MS	PI-MS^2^
Anthraquinone	1	8-*O*-Methylchrysophanol	5.51	nd^b^	nd	291.1 [M + Na]^+^	139.1
	2	Physcion-8-*O*-β-d-glucoside^a^	7.51	445.1 [M–H]^−^	283.1	nd	nd
	3	Rhein-8-*O*-β-d-glucoside^a^	7.73	445.1 [M–H]^−^	239.1	nd	nd
	4	Chrysophanol-8-*O*-(6′-*O*-malonyl)-glucoside	7.88	567.2 [M–H]^−^	169.1	nd	nd
	5	Physcion-8-*O*-β-d-(6′-acetyl)-glucoside	8.30	487.1 [M–H]^−^	283	nd	nd
	6	Emodin-8-*O*-β-d-glucoside^a^	8.68	431.3 [M–H]^−^	269.1	433.1 [M + H]^+^	271.1
	7	Chrysophanol-*O*-glucoside	8.81	415.1 [M–H]^−^	253.1	nd	nd
	8	Emodin-*O*-(6′-*O*-acetyl)-glucoside	9.06	473.1 [M–H]^−^	269.1	nd	nd
	9	Emodin-8-*O*-(6′-*O*-malonyl)-glucoside	9.07	517.1 [M–H]^−^	473.1	nd	nd
	10	Chrysophanol-*O*-(6′-acetyl)-glucoside	9.46	457.1 [M–H]^−^	253.1	481.1 [M + Na]^+^	255.1
	11	Rhein-1-*O*-(*O*-acetyl)-glucoside	9.85	487.1 [M–H]^−^	239.1	489.1 [M + H]^+^	241.1
	12	Citreorosein	10.21	285.1 [M–H]^−^	211.1	287.1 [M + H]^+^	213.1
	13	Aloe emodin^a^	10.33	269.2 [M–H]^−^	240.1	271.1 [M + H]^+^	242.1
	14	Rhein^a^	11.45	283.2 [M–H]^−^	239.1	nd	nd
	15	Emodin^a^	11.72	269.2 [M–H]^−^	225.1	271.1 [M + H]^+^	227.1
	16	Chrysophanol^a^	13.10	253.2 [M–H]^−^	225.1	277.1 [M + Na]^+^	227.1
	17	Physcion^a^	13.28	283.3 [M–H]^−^	240.1	nd	nd
	18	Emodin bianthrones B + C_3_H_2_	13.54	547.1 [M–H]^−^	254.1	nd	nd
	19	Emodin bianthrones B	14.03	509.1 [M–H]^−^	254.1	nd	nd
	20	Emodin bianthrones B + CH_2_COOH	14.16	568.1 [M–H]^−^	254.1	nd	nd
	21	Rheidin A	16.13	523.1 [M–H]^−^	254.1	nd	nd
Flavonoids	22	Kaempferol	5.29	285.1 [M–H]^−^	211.1	287.1 [M + H]^+^	213.1
	23	Quercetin-3-*O*-[acetyl-galactoside]	5.55	nd	nd	465.1 [M + H]^+^	345.1
	24	12-(3-Hydroxyethyl)-cytisine	6.10	nd	nd	235.1 [M + H]^+^	189.1
	25	Myricetin-3-*O*-glucoside	6.25	nd	nd	481.1 [M + H]^+^	319.1
	26	Isovitexin	6.54	nd	nd	433.1 [M + H]^+^	283.1
	27	Hyperin^a^	6.66	nd	nd	465.1 [M + H]^+^	303.1
	28	Quercetin^a^	6.67	301.1 [M–H]^−^	151.1	303.1 [M + H]^+^	153.1
	29	Quercetin-3-*O*-[6′'-(3-hydroxy-3-methylglutaroyl)-galactoside]	6.85	607.1 [M–H]^−^	303.1	609.1 [M + H]^+^	303.1
	30	2′'-*O*-galloylvitexin	6.85	nd	nd	585.1 [M + H]^+^	313.1
	31	Quercetin-3-Glucuronide + CH_2_	7.76	nd	nd	477.1 [M + H]^+^	303.1
	32	Luteolin^a^	7.00	nd	nd	287.1 [M + H]^+^	153.1
	33	4′-Hydroxy-5,7-dimethoxyflavanone	7.17	nd	nd	463.1 [M + H]^+^	181.1
	34	Aphonol	7.49	nd	nd	433.1 [M + H]^+^	287.1
	35	Rutin^a^	6.41	nd	nd	611.1 [M + H]^+^	303.1
	36	Naringenin	9.82	nd	nd	273.1 [M + H]^+^	153.1
	37	Apigenin^a^	13.31	269.1 [M–H]^−^	151.1	nd	nd
	38	Pinobaksin	14.45	nd	nd	255.1 [M + H]^+^	153.1
Stilbenes	39	Resveratrol-glucoside	7.30	389.1 [M–H]^−^	227.1	nd	nd
	40	Rhapontigenin-(6′-*O*-acetyl)-glucoside	7.40	461.1 [M–H]^−^461	257.1	nd	nd
	41	Rhapontigenin	7.60	257.1 [M–H]^−^257	215	nd	nd
	42	Rhapontigenin-glucoside	7.65	419.1 [M–H]^−^419	257.1	nd	nd
	43	Resveratrol-(6′-*O*-galloyl)-glucoside	7.83	541.1 [M–H]^−^541	313.1	nd	nd
	44	Piceatannol-*O*-β-d-glucoside	8.52	405.1 [M–H]^−^405	243.1	nd	nd
Galloyl esters	45	Galloyl-glucose	2.76	331.1 [M–H]^−^331	125.1	nd	nd
	46	Gallic acid^a^	3.11	169.1 [M–H]^−^169	125.1	nd	nd
	47	di-*O*-galloyl-glucose	5.23	483.1 [M–H]^−^483	169.1	nd	nd
	48	*p*-Coumaroyl-*O*-galloyl-glucose	6.20	477.1 [M–H]^−^477	313.1	nd	nd
	49	tri-*O*-Galloyl-glucose	6.27	635.1 [M–H]^−^635	465.1	nd	nd
	50	*p*-Coumaroyl-di-*O*-galloyl-glucose	7.67	629.1 [M–H]^−^629	169.1	nd	nd
	51	*p*-Coumaroyl-*O*-*p*-hydroxybenzoyl-galloyl-glucose	8.02	597.1 [M–H]^−^597	433.1	nd	nd
	52	Cinnamoyl-*O*-galloyl-glucose	8.08	461.1 [M–H]^−^461	125.1	nd	nd
	53	di-Coumaroyl-*O*-galloyl-glucose	8.36	623.1 [M–H]^−^623	459.1	nd	nd
	54	Coumaroyl-*O*-feruloyl-*O*-galloyl-glucose	8.49	653.1 [M–H]^−^653	489.1	nd	nd
	55	Cinnamoyl-di-*O*-galloyl-glucose	8.63	613.1 [M–H]^−^613	169.1	nd	nd
	56	Coumaroyl-*O*-cinnamoyl-*O*-galloyl-glucose	9.44	607.1 [M–H]^−^607	443.1	nd	nd
Tanins	57	Catechin-glucoside	4.93	nd	nd	453.1 [M + H]^+^	139.1
	58	(−)-Epicatechin gallate	7.17	441.1 [M–H]^−^	169.1	nd	nd
	59	(+)-Catechin^a^	7.19	289.1 [M–H]^−^289	255.1	nd	nd
Naphthylenes	60	Torachrysone-8-*O*-β-d-glucoside	6.19	nd	nd	409.1 [M + H]^+^	247.1
Alkaloids	61	Ephedrine	4.59	nd	nd	166.1 [M + H]^+^	77.1
Coumarin	62	3(2′-Chlorophenyl)-7-ethoxycoumarin	7.14	nd	nd	463.1 [M + H]^+^	217.1

^a^Identification was confirmed through comparison with standards.

^b^*nd* not determined.

Compounds (22–38) were unambiguously identified as flavonoids. Compounds 27 and 35 were identified as quercetin derivatives, which were deduced to have the same aglycone ion m/z 303.1 observed by PI-MS^2^, in agreement with ions [M + H]^+^ at m/z 465.1 and [M + H]^+^ at m/z 611.1, respectively, with the molecular weight 464.1 and 609.1 was fragmented to the ion [M + H-162 Da]^+^ at m/z 303.1, and [M + H-308 Da]^+^ at m/z 303.1, respectively, indicating loss of glucoside and rhamnose, and these compounds identified as hyperfine (quercetin-3-*O*-glucoside) and rutin (quercetin-3-*O*-rhamnose) which was validated with authentic standard. Totally, 17 flavonoids were identified based on PI-MS and/or NI-MS determined to have the molecular weight and characteristic alycone ion at PI-MS^2^ and/or NI-MS2 as showed in Table 1.

Compounds (39–44) were identified as stilbens. Compounds 42 were determined to have the molecular weight 420.1 with ion [M–H]^−^ 419.1, with a characteristic ion [M–H-162 Da]^−^ at m/z 257.1, indicating loss of glucoside, it was unequivocally identified as rhapontigenin-glucoside, furthermore, based on characteristic ions of the same aglycone, compounds 40 and 41 were also identified as rhapontigenin derivatives, which has also been previous identified in rhubarb^31^.

Compounds (45–56) were identified as galloyl esters. Compound 47 exhibited the deprotonated ion [M–H]^−^ at m/z 483.1 and major ion characteristic fragment ion at m/z 169.1 and was deduced to be di-*O*-galloyl-glucoside. Among them, galloyl esters series compounds had been previously identified in rhubarb^29^. Compounds (57–59) were identified as tannins, catechin (compound 59) was unequivocally identified in reference to the authentic standards.

Compound 60 was tentatively identified as torachrysone-8-*O*-glucoside, with a molecular weight of 410.1 at m/z 409.1 [M–H]^−^. Fragment ions [M-162 Da]^−^ at m/z 247.1 corresponded to the loss of glucoside. According to the literature^29^, torachrysone-8-*O*-glucoside has been previously isolated from rhubarb. Compounds 61 and 62 were tentatively identified as ephedrin and 3(2′-Chlorophenyl)-7-ethoxycoumarin based on the characteristic ions, which were also reported previously in rhubarb^30^. Collectively, 62 compounds were determined from the retention times, fragment ion information from MS and MS–MS, available standards, and previous literatures, as shown in Table 1.

**Figure 1:**
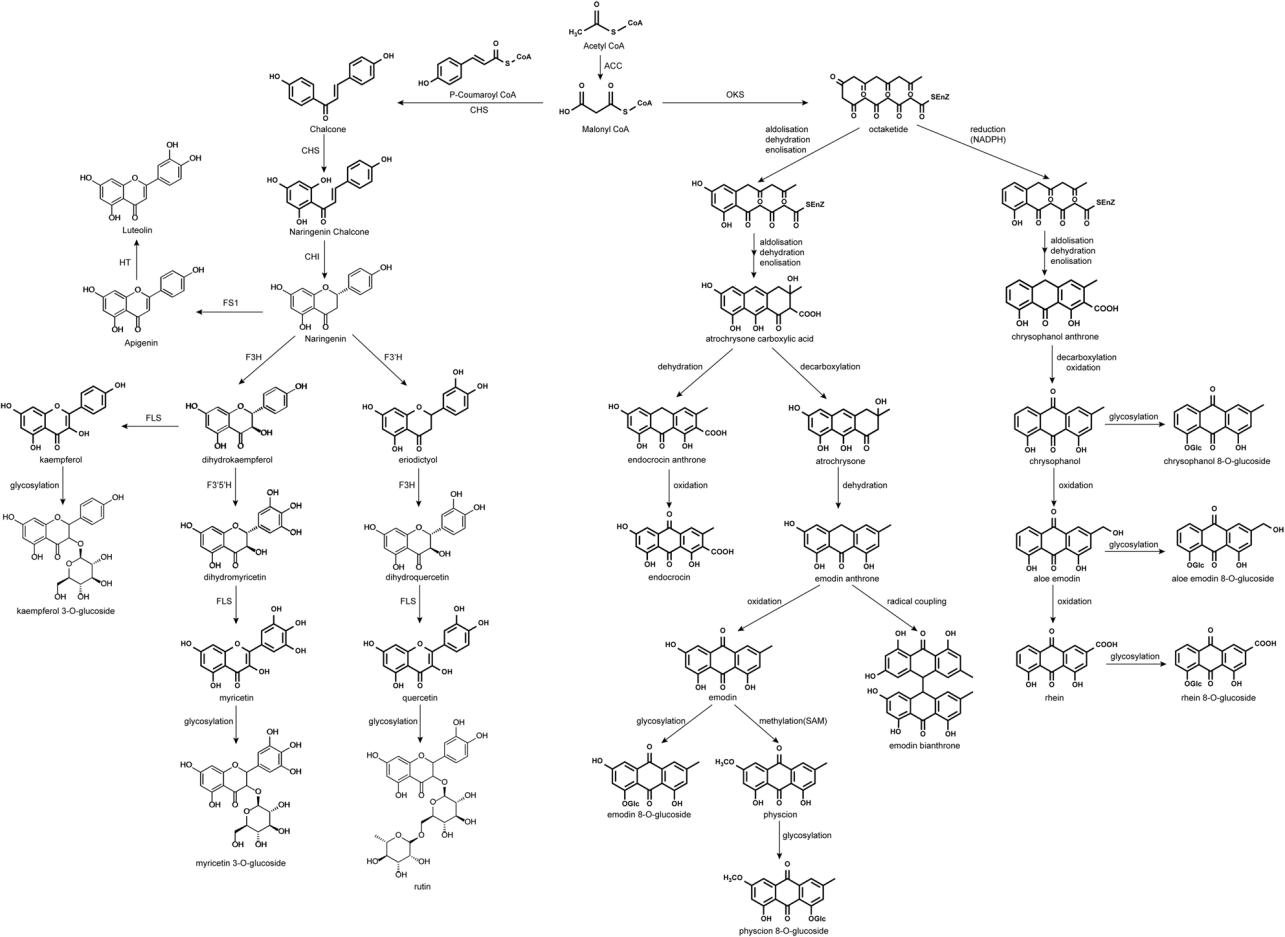
Putative anthraquinone and flavonoid biosynthetic pathways in rhubarb.

The main routes of anthraquinone biosynthesis have been previously reported, where there are two main distinct biosynthetic pathways leading to anthraquinones in higher plants, including the polyketide pathway and chorismate/*o*-succinylbenzoic aid pathway^7^. However, the anthraquinone biosynthetic pathway in rhubarb is not clear and the annotation of anthraquinone metabolic pathway is still absent. Besides, anthraquinones and flavonoids are the main pharmacologically active ingredients in rhubarb. As the flavonoid biosynthetic pathway belongs to the polyketide pathway, several correlations between the anthraquinone and flavonoid biosynthetic pathways may exist. Taking into consideration the main ingredients in rhubarb and the results of previous studies, a putative rhubarb biosynthetic pathway for anthraquinones and flavonoids was proposed^32,33^, as shown in Fig. 1. It can be seen from the pathway that polyketide backbone formation, methylation, and glycosylation exist both in anthraquinones and flavonoids, suggesting that anthraquinone and flavonoid synthesis may share the same functional enzyme genes.

### Large-scale detection, identification and quantification of target metabolites using dMRM-MS/MS

A large-scale quantification method was carried out to construct a tandem mass spectrometry (MS2) spectral tag library from target metabolite detection using paired ions. A dynamic multiple-reaction-monitoring (MRM) mode under unit mass-resolution was used for simultaneous quantitative measurement of all the detected metabolites. It is the first time that the 62 metabolic compounds were simultaneously quantitatively identified and qualitatively assessed in rhubarb, and among which, only gallic acid and piceatannol-*O*-β-glucoside did not show any significant difference between the two species. The remaining 60 compounds exhibited significant differences (*P* < 0.05), and among which, the levels of 17 compounds in RPL was significantly higher than that in ROB, whereas it was the opposite for the remaining 43 compounds. Regarding anthraquinones, 7 free anthraquinones and 14 anthraquinone conjugates were simultaneously identified and quantified in the tested samples. Among the seven free anthraquinones in RPL, the levels of four substances, including 8-*O*-methyl chrysophanol, chrysophanol, citreorosein, and physcion, were significantly lower than that observed in ROB, while the level of aloe-emodin, emodin, and rhein in RPL was significantly higher. Of the 14 anthraquinone conjugates, 8 compounds in RPL had contents that were significantly lower than that detected in ROB.

**Figure 2:**
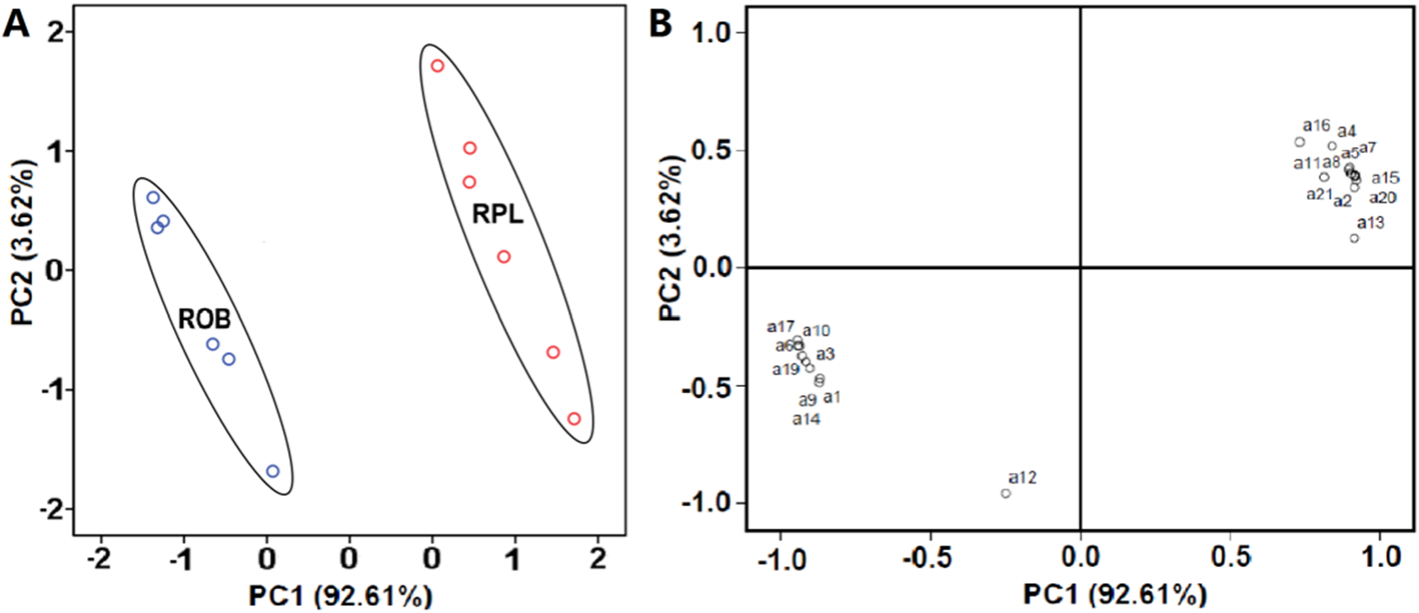
Positions of principal component analysis (PCA) scores (PC1; PC2) of the detected compounds in the two rhubarb species. Notes: Percentages in the parentheses represent principal component variance. The number in (**B**) represent the compound number, which corresponds to the same compounds in Table 1. (**A**) Scatter plot of PCA scores; (**B**) loadings plot of PCA.

Principal component analysis was used to examine the quantitative data of the anthraquinone compounds in the two species of rhubarb. The variance contribution rate of the first eigenvalue was 92.61%, the second eigenvalue was 3.62%, and the accumulative contribution rate of the first two factors was 96.23%. The six replicates of ROB and RPL leaves were clustered on the left and right sides of PC1, respectively, indicating a good correlation between biological repeats, showing a significant difference between the two kinds of rhubarb (Fig. 2A). Besides, a significant difference was observed in the anthraquinone content between the two rhubarb species (Fig. 2B).

### Method validation

The calibration curves showed good linearity for all available standards. The standard solutions were detected through chromatography until the signal-to-noise ratio corresponded to 3 and 10, for the limit of detection and limit of quantitation, respectively. Metabolic quantification precision was studied by examining the repeatability and intermediate precision for all the compounds.

### *Sequencing and *de novo* transcriptome assembly*

RNA sequencing was performed using Illumina paired-end sequencing technology. A total of 193,873,248 Illumina raw reads were obtained (Table 2). After removing the adaptor sequences, ambiguous nucleotides and low-quality sequences, approximately 185 million clean reads were kept for subsequent analysis. These reads were then assembled de novo using the Trinity tool^34^. After eliminating the repeated sequences, as well as those shorter than 200 bp, the assembly of clean reads resulted in 180, 689 transcripts and 90,242 unigenes. These unigenes ranged from 201 to 14,669 bp, with a mean length of 1052 bp, median length of 706 bp and N50 length of 1556 bp (Figure S2).

**Table 2:** Summary of de novo sequence assembly for rhubarb leaves.

Sample	Raw reads	Clean reads	Clean bases	Error (%)	Q20 (%)	Q30 (%)	GC content (%)
ROB_1^a^	51367574	48795386	7.32G	0.02	95.65	89.28	47.98
ROB_2	49305010	46904390	7.04G	0.02	95.78	89.56	48.19
RPL_1^b^	45103294	43417756	6.51G	0.02	96.93	92.27	48.98
RPL_2	48097370	45563138	6.83G	0.02	95.42	88.84	47.79

^a^ROB, *R. officinale Baill.*; ^b^RPL, *R. palmatum *L. Samples 1 and 2 represent two biological repeats.

### Annotation of unigenes

A homology search in the Nr database was conducted for all unigenes using the BLAST program^35^ with a cutoff E-value < 10^−5^ to determine their quality_._ The best aligning results were selected to annotate the unigenes. The results showed that 56% of the aligned sequences (50,558) exhibited significant homology with entries in the Nr database (E-value < 10^−5^), whereas > 75% of these matched sequences showed an E-value < 10^−15^ (Figure S3). Based on the BLAST similarity distribution, 32.5% of the aligned sequences exhibited alignment identities > 80% (Figure S3). These 90,242 non-redundant unigenes were screened for similarity in seven public databases, namely, Nr, Nt, Swiss-Prot, Kyoto Encyclopedia of Genes and Genomes (KEGG), gene ontology (GO), KOG, and Pfam. The annotation results showed that 56.05% (50,588 unigenes) had hits in the Nr database, followed by the Swiss-Prot database (45.15%, 40,753 unigenes). In total, there were 57,239 unigenes (63.42%) annotated in at least one of the seven databases, with 8819 unigenes (9.77%) appearing in all seven databases including those annotated in Nr, Nt, KEGG, Swiss-Prot, Pfam, and GO databases.

### Functional classification of unigenes

**Figure 3:**
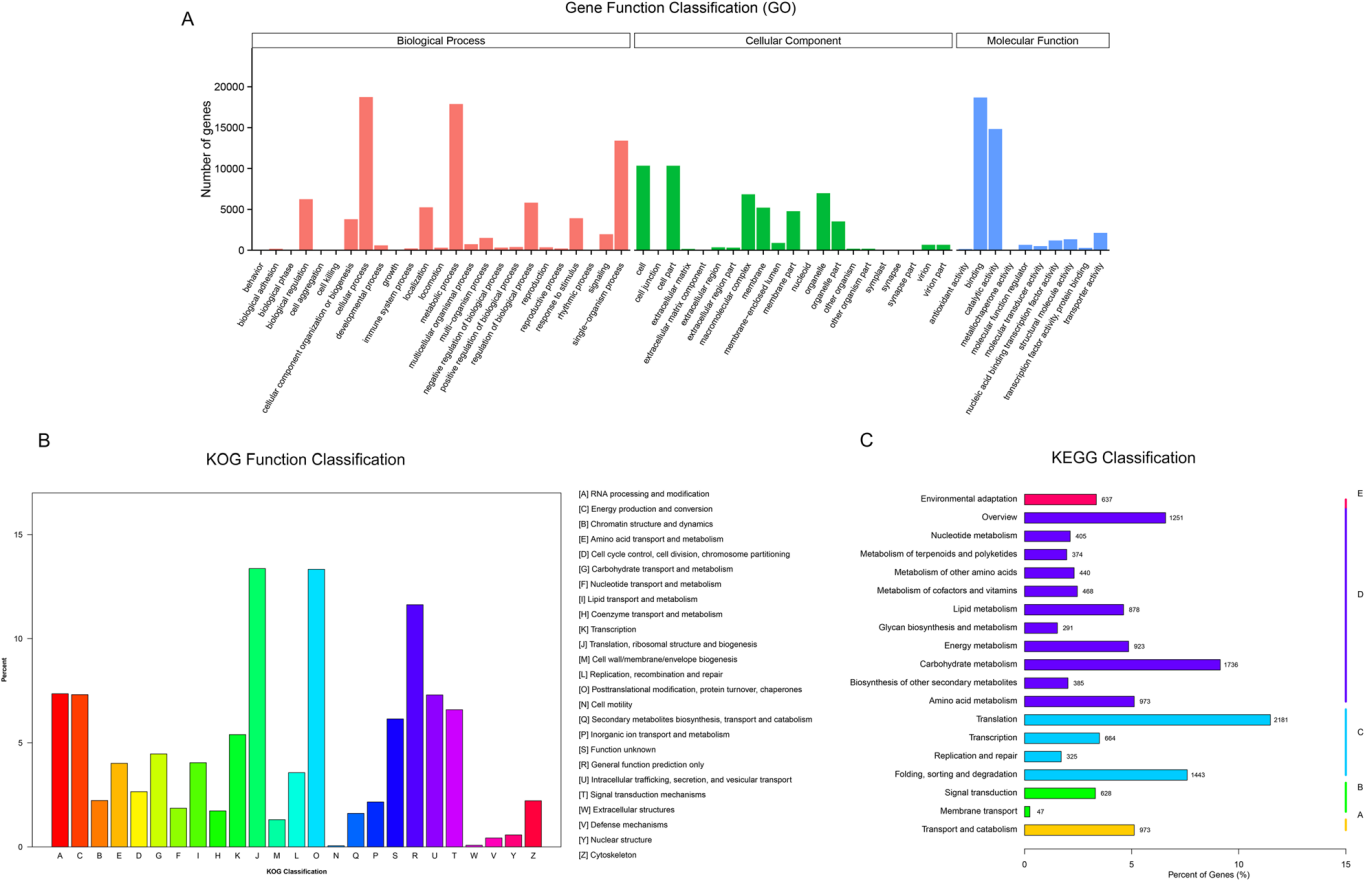
Functional categorization of unigens determined from three biologically processed classifications. (**A**) Gene Ontology (Go). (**B**) EuKaryotic Orthologous Groups (KOG). (**C**) Kyoto Encyclopedia of Genes and Genomes (KEGG).

GO was used to classify the functions of the predicted rhubarb unigenes^36^. In total, 39,567 unigenes with BLAST matched with known proteins were classified into 56 functional groups under three main categories (biological process, cellular component, and molecular function) using 1,660 functional terms (Fig. 3a). Majority of the unigenes were assigned to “biological processes” (488,916), followed by “cellular components” (145,217), and “molecular functions” (98,389). Within biological processes, metabolic process (242,663) and cellular process (108,192) were the most dominant terms. Under the category of cellular components, cell part (44,594) and organelle (33,420) had the highest representation. For molecular functions, the most represented were binding activity (72,789) and catalytic activity (18,096).

All unigenes were aligned with the KOG database in which orthologous genes were classified according to possible functions. A total of 16,395 unigenes were clustered into 25 KOG classifications (Fig. 3b, Table 2), in which the cluster of “translation”, “ribosomal structure” and “biogenesis” (2766) was the largest group, followed by “post-translational modification”, “protein turnover”, and “chaperon” (2112), as well as general function prediction of function only (1801).

The KEGG pathway database is a knowledge base for the systematic analysis of gene functions in terms of gene and molecule networks in cells^37^. Based on KEGG, 21,691 unigenes were assigned to 130 pathways (Table 2, Fig. 3). The pathways involving the largest number of translations were carbohydrate metabolism (3632, 16.74%) and translation (3117, 14.37%). In contrast, membrane transport (59, 0.27%) was the smallest group (Fig. 3c).

### Identification of DEGs

The Pearson’s correlation coefficient (*r*^2^) of gene expression between RPL and ROB was calculated to further analyze the gene expression levels. The results showed that the Pearson’s correlation coefficient (*r*^2^) among samples was relatively high in ROB_1 versus ROB_2 (*r*^2^ = 0.880) and RPL_1 versus RPL_2 (*r*^2^ = 0.867), whereas it was low in ROB versus RPL (from 0.527 to 0.576), indicating that there is a large number of DEGs between ROB and RPL. In addition, the read counts of every gene obtained through Hi-seq in all four RNA-sequencing samples (two biological replicates in ROB and RPL, respectively) were normalized and analyzed using the DE-Seq package. Finally, 19,242 genes were detected as significantly differentially expressed between ROB and RPL. Taking the analysis of the annotation results together, 121 genes appeared to be related to the flavonoid synthesis pathway, and 38 genes were significantly differentially expressed between ROB and RPL. Among the 38 DEGs, 17 were tentatively considered to be related with the anthraquinone synthesis pathway. There were four DEGs (cluster-19180.1, cluster-14354.16291, cluster-14354.38644, and cluster-14354.40715) annotated as polyketide synthase genes, which may catalyze the initial synthesis of anthraquinone to form a phenyl ring skeletal structure; there were seven DEGs (cluster-14354.36512, cluster-14354.38156, cluster-14354.46426, cluster-14354.26346, cluster-14354.3016, cluster-14354.37142, and cluster-14354.3014) annotated as caffeoyl-CoA methyltransferase gene, which may catalyze anthraquinone methylation; and there were six DEGs (cluster-14354.44810, cluster-14354.46428, cluster-14354.44811, cluster-14354.53295, cluster-14354.53303, and cluster-14354.58524) annotated as UDP glycosyltransferase gene, which may catalyze free anthraquinone binding with glycosyl groups to generate anthraquinone conjugates.

### Combined analysis of RNA-sequencing and metabolomics data

**Figure 4:**
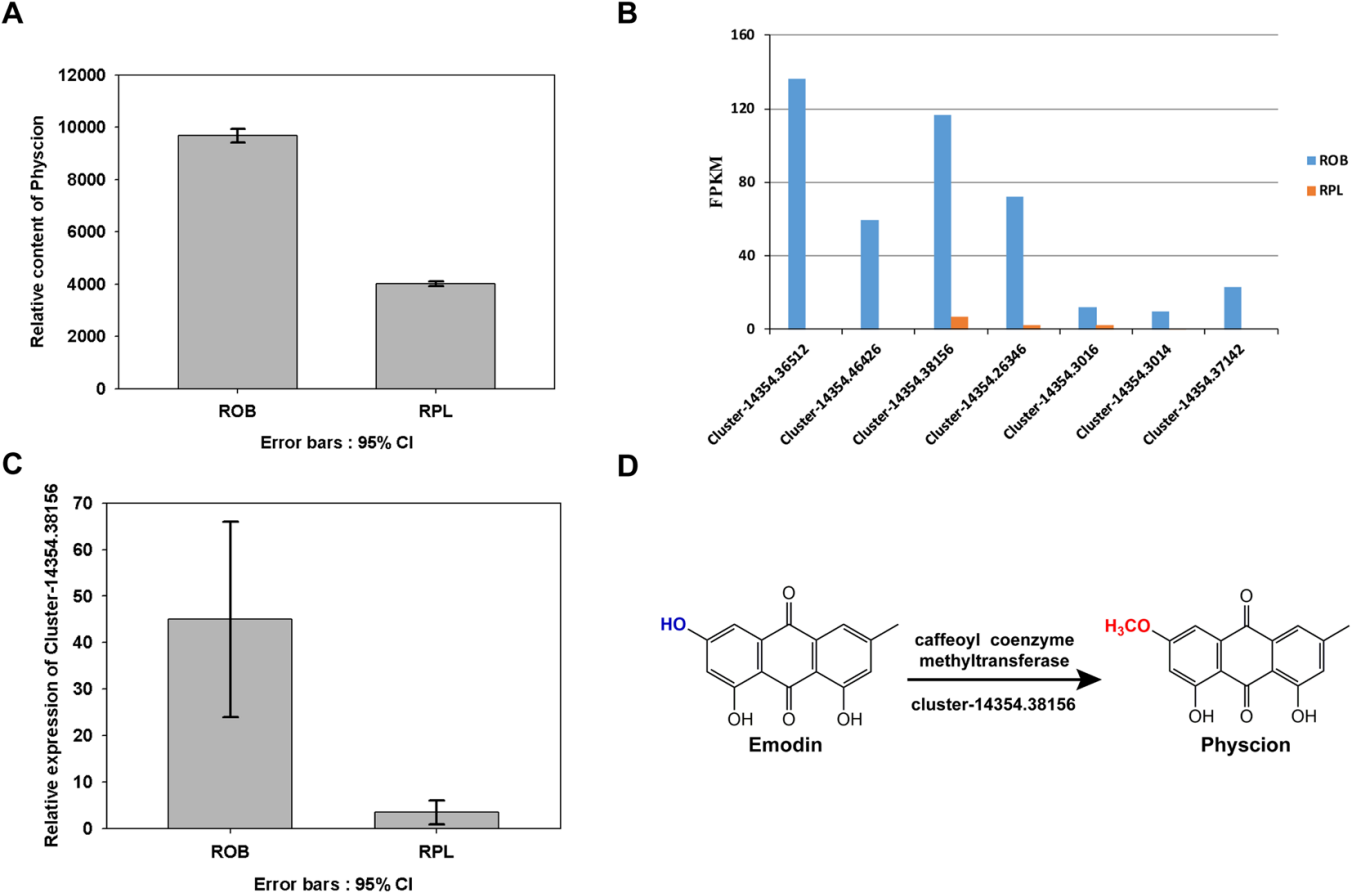
Relative contents of physcion in the two rhubarb species (**A**) (relative content ± SD), different expression levels of the seven selected genes (**B**), related gene relative expression in the two rhubarb species (**C**) (relative content ± SD), and candidate gene cluster-14354.38156 may lead to conversion of emodin to physcion (**D**).

The content of 21 anthraquinones in ROB and RPL and the 38 DEGs expressions annotated by the flavonoid synthesis pathway were analyzed through constructing a heat map (Figure S4) to further investigate the molecular mechanism of the secondary metabolite varieties in different rhubarb species. Correlation analysis between DEGs expression levels and compound peak intensities showed that all the DEG expression levels had high correlation with the anthraquinone peak area (P < 0.05), suggesting that these DEGs contributed to the anthraquinone biosynthetic pathway. It has been reported that emodin was methylated by methyltransferase to form physcion^32^. According to the results of the metabolomics analysis, emodin level in ROB was slightly lower than that observed in RPL. In contrast, physcion content in ROB was approximately 2.4-fold higher than that observed in RPL (Fig. 4A). We then searched possible biosynthetic genes that may have contributed to this difference, since the physcion level was different in ROB and RPL, and a significant difference was observed in the correlation analysis between the peak intensity of physcion and all the seven DEGs annotated as caffeoyl-CoA methyltransferase genes. The results showed that the expression of four DEGs (cluster-14354.38156, cluster-14354.26346, cluster-14354.3016, and cluster-14354.3014) in ROB was higher than that in RPL (Fig. 4B).

We used RT-qPCR to quantify the transcript levels of the four genes obtained from the aforementioned analysis. The results showed that the expression of the cluster-14354.38156 in the ROB was approximately 14-fold higher than that observed in RPL (Fig. 4C). This finding was consistent with the difference detected by RNA-seq gene expression. Therefore, cluster-14354.38156 may catalyze the metabolic reaction of emodin 3 methylation to produce physcion (Fig. 4D).

## Discussion

Natural products from plants and animals are important economic commodities, as these are widely used as food and dietary supplements. Comprehensive metabolomics analysis is important not only for pharmacologic valuation, but also for quality control. For products obtained from species present in different countries, the transcriptome approach can provide a reliable means of gene discovery, in combination with accurate quantitative analysis of metabolites. In total, 62 compounds were quantitated using dynamic MRM mode under unit mass-resolution, among which 21 anthraquinone compounds have upstream or downstream biosynthetic relations, presumably having different medicinal values. A comprehensive and systematic metabolomics analysis is of great significance to utilizing the medicinal value of the characteristic compounds in the two rhubarb species.

The combination of functional gene expression and metabolomics data is a useful approach based on the principle that genes and metabolites involved in the same metabolic processes exhibit identical or similar variations, but the lack of genome information usually makes analysis difficult. Thus, transcriptome analysis provides a valuable way to simultaneously investigate functional genes and metabolites. The differences in metabolite content among different plant varieties or tissues can be determined through metabolomics analysis, whereas the differences in gene expression were determined through transcriptome sequencing. Taken together, DEGs involved in metabolic pathways are possible genes functioning in the synthesis of metabolites. In the present study, we generated 180,689 transcripts and 90,242 unigenes through de novo transcriptome assembly. GO and KEGG pathway analyses characterized the unigenes, resulting in 21,691 unigenes with metabolic pathway annotations. A total of 121 genes were annotated in the flavonoid synthesis pathway, and 38 of them showed significant differences in expression levels between RPL and ROB. The metabolomics results showed that significant differences were observed among the 60 metabolites, except for gallic acid and piceatannol-*O*-β-glucoside. Combining the metabolome and transcriptome results, 17 DEGs were considered to be related with the anthraquinone biosynthetic pathway. This included four DEGs annotated as polyketide synthase genes, seven DEGs annotated as caffeoyl-CoA methyltransferase genes, and six DEGs annotated as UDP glycosyltransferase genes. Additionally, there was a significant correlation between anthraquinone peak intensity and DEGs expression level (P < 0.05), validating that these DEGs contributed to the anthraquinone biosynthetic pathway. Further, RT-qPCR results showed that the expression of the cluster-14354.38156 in ROB was approximately 14-fold higher than that observed in RPL, which may participate in the *O*-methylation of emodin to produce physcion.

In the present study, we first systematically accessed the metabolic diversity in rhubarbs. Combining metabolomics and transcriptomic analyses, 17 DEGs were considered to contribute to the anthraquinone biosynthetic pathway, and a possible gene candidate that could catalyze the methylation of the hydroxyl group at the 3-position of emodin to produce physcion was predicted. Our study showed that the combined analysis of transcriptome and metabolome can indeed help in the search for enzyme genes involved in metabolite biosynthesis, providing a useful resource for further studies on metabolites in rhubarb species.

## Methods

### Plant materials and chemicals

The two rhubarb species used in the study, *R. palmatum L*. (RPL) and *R. officinale Baill.* (ROB), were cultivated under the same condition at the wild breeding base of medicinal materials in Enwei Plateau, Kangding, Sichuan, China. The identification of RPL and ROB from the collected rhizome specimens was conducted by Professor Li Xiang at the Institute of Chinese Materia Medica, China Academy of Chinese Medical Science, Beijing, China. Fresh leaves were frozen in liquid nitrogen, and then stored at − 80 °C for further analysis. Gradient grades of methanol, acetonitrile, and acetic acid were purchased from Sigma-Aldrich (St. Louis, MO, USA). Formic acid (eluent additive used for HPLC–MS analysis) used was of MS grade (CNW, Germany). The remaining analytical-grade chemicals were obtained from Beijing Chemical Factory (Beijing, China). Pure standards of major anthraquinones, namely, rhein (No. 0757-9804), emodin (No. 0756-9707), aloe-emodin (No. 110795-201007), chrysophanol (No. 110796–201319), physcion (No. 0758-9402) (> 98%), physcion-8-*O*-β-d-glucoside (No. 150106), rhein-8-*O*-β-d-glucoside (No. BCTG-0129), Emodin-8-*O*-β-d-glucoside (No. BCTG-0130), rutin (No. 100080-200707), apigenin (No. BCY-0516), hyperin (No. 111521-201205), quercetin (No. 0081-9304), luteolin (No. BCY-000544) (standard of flavonoid), catechin (No. 110877–201203), gallic acid (No. 110831-201204), and saikosaponin A (internal standard, IS) were purchased from the National Institute for the Control of Biological and Pharmaceutical Drugs (Beijing, China). Emodin-8-*O*-β-d-glucoside (> 97.6%) was obtained from the laboratory where the study was conducted^17^.

### Sample preparation and extraction

Accurately weighed 100 g rhein fresh leaf tissue was crushed into a fine powder using a mixer mill with a zirconia bead for 1.5 min at 30 Hz. The samples were extracted overnight at 4 °C with 1.0 ml pure methanol (or 70% aqueous methanol) containing 0.1 ppm lidocaine to absorb lipid-soluble extracts. Then, 0.4 ml of each extract was mixed and filtered (SCAA-104, 0.22 µm pore size) before LC–MS analysis.

### ESI-Q-TOF–MS/MS

The analytical conditions were as follows: UPLC: column, Agilent Eclipse-Plus C_18_ column (pore size 1.8 µm, length 2.1 mm × 50 mm); solvent system, water (0.1% formic acid), acetonitrile (0.1% formic acid); gradient program, 100:0 v/v at 0 min, 5:95 v/v, at 20.0 min, 5:95 v/v at 22.0 min, 95:5 v/v at 22.1 min, and 95:5 v/v at 28 min; flow rate, 0.2 mL/min; temperature, 40 °C; injection volume: 1 µl.

Full time-of-flight (TOF) scans of UPLC effluents were acquired in the mass range of 50–1000 using an Agilent 6540-mass time-of-flight (Q-TOF) mass spectrometer equipped with a Dual ESI electrospray ion source in both positive and negative modes. The ESI source operation parameters operating in a positive ion mode and the ESI–MS conditions are as follows: gas temperature: 325 °C, gas flow: 5 L/min, nebulizer: 35 psig, sheath gas temp: 350 °C, collision energy voltage: 20 V (ESI+) and 30 V (ESI+), as well as 40 V (ESI+). Internal references (purine and HP-0921) were adopted to modify the measured masses in real time, and the reference masses in the positive ion mode were at m/z 121.0509 and 922.0098, whereas they were at m/z 119.0363 and 1033.9881 in the negative ion mode.

### ESI-Q-TRAP-MS/MS

A triple quadrupole-linear ion trap mass spectrometer (Q-trap), Q-TRAP 5500 LC–MS/MS system, equipped with an ESI-Turbo Ion-Spray interface, was operated in a positive and negative mode using the Analyst 1.6.2 software (AB Sciex). The ESI source operation parameters were as follows: source temperature 500 °C, ion spray voltage (IS) 5500 V; ion source gas I (GS I), gas II (GS II), collision gas (CAD), and curtain gas (CUG) were set at 50, 50, 30, and 20 psi, respectively. Collision energy was also set at 20, 30 and 40 eV. Instrument running and mass calibration were performed with 1, 10 and 100 mol/L polypropylene glycol solution in trap and LIP modes, respectively. The MIM scan that served as a survey scan to trigger information-dependent acquisition of the EPI scan model, MIM-EPI, was carried out to screen metabolite ions isolated in Q1 minimal CE (5 eV). The corresponding Q3 of the same metabolites were monitored through two ways. Firstly, a modified MIM-EPI scan was adapted, in which Q1 and Q3 were set from 50.1 to 1000.0 Da, and the mass step was 0.2 Da. Second, another targeted scan was also acquired for the paired Q1 and Q3. We monitored each MIM-EPI experiment with 60 MIM transitions, and the product ions of each metabolite ion were scanned from 50.1 to 1000.0 Da in Q3, and the total cycle time for one scan was approximately 2.0 s.

### QqQ-experiment

The sample extracts were analyzed using UPLC-ESI-QqQ-MS (Agilent 1290 and 6460 triple-quadrupole mass spectrometry series, Agilent Corporation, CA, USA). Quantitative data was acquired by QqQ scanning using an improved Dynamic MRM experiment, with collision gas (nitrogen) set to 5 psi. Improvement of paired Q1–Q3 mass, CE, retention time, and fragment for individual MRM transitions were done based on the EPI experimental and targeted scans. Each MRM transition was obtained with a 10-ms dell time and 10-ms pause time. The MS conditions in the positive mode were as follows: HV voltage, 4,000 kV; capillary, 7 µA; nozzle voltage, 500 V; delta EMV, 300 V; gas flow, 5 L/min; gas temperature, 400 °C; and sheath gas flow, 11 L/min. Collision energy was optimized based on the standards. Helium was used as the collision gas for collision-induced dissociation. Quantification was done using the MRM mode under unit mass-resolution conditions.

The t-test was used for the quantitative analysis of compound results. Principal component analysis was performed on the metabolic data for both rhubarb species.

### RNA extraction, library construction, and sequencing

Fresh leaves of the two rhubarb species were individually ground into powder in liquid nitrogen, and each sample was with two biological replicates. Total RNA was isolated using the RNA prep Pure Kit (For Plant TIANGEN Biotech, Beijing, China), and RNA degradation and contamination were determined using 1% agarose gels. RNA purity was verified using the Nano-Photometer spectrophotometer (Implen, CA, USA). RNA concentration was measured using the Qubit RNA Assay Kit with a Qubit 2.0 fluorometer (Life Technologies, CA, USA). RNA integrity was assessed using the RNA Nano 6000 Assay Kit on the Agilent Bioanalyzer 2100 system (Agilent Technologies, CA, USA).

A total amount of 1.5 μg RNA per sample was used as the input material for the RNA sample preparations. Sequencing libraries were generated using NEB Next Ultra RNA Library Prep Kit for Illumina (NEB, USA) according to the manufacturer’s instructions, and the index codes were added to attribute sequences to each sample. The clustering of the index-coded samples was performed on a cBot Cluster Generation System using TruSeq PE Cluster Kit v3-cBot-HS (Illumina), according to the manufacturer’s instructions. After cluster generation, the library preparations were sequenced using an Illumina Hi-seq 2500 platform and paired-end reads were generated.

### Sequence read mapping, assembly, and annotation

Raw data in FASTQ format were first processed through in-house Perl scripts. Reads containing adapter sequences, Ns, and low-quality reads were excluded from the raw data. The remaining reads were kept as clean data. Subsequently, the Q20, Q30, and GC content of the clean data were calculated. All downstream analyses were based on high-quality clean data.

The FASTQ files from all four samples were pooled for subsequent assembly. Transcriptome assembly was accomplished using the Trinity software^26^ with all parameters set to default. All assembled contigs were then clustered to unigenes.

Gene function was annotated based on seven databases, namely, Nr (National Center for Biotechnology Information (NCBI) non-redundant protein sequences), Nt (NCBI non-redundant nucleotide sequences), Pfam (protein family)^38^, EuKaryotic Orthologous Groups (KOG)/ Clusters of Orthologous Groups of proteins, Swiss-Prot (a manually annotated and reviewed protein sequence database), KEGG Ortholog database^39,40^, and GO. Data for each sequenced library were analyzed using the BLAST program with a cutoff E-value of 10^−5^.

### Differential expression analysis of unigenes

The levels of gene expression in each sample were estimated using RSEM^23^. Clean data were mapped back onto the assembled transcriptome, and a read count for each gene was obtained using Hi-sequencing. Differential expression analyses of two conditions/groups were performed using the DE-Seq R package (1.10.1)^14^. Count data generated using RSEM were used as input, and two groups, for example, two ROB samples and two RPL samples, were normalized and further used for differential expression analysis using a DE-Seq dataset from Matrix and DE-Seq functions in the DE-Seq package. DE-Seq provided statistical routines for determining differential gene expression using a model based on negative binomial distribution. The resulting *P* values were adjusted, and the genes with adjusted *P*-values < 0.05 (according to DE-Seq) were identified as differentially expressed.

### Combined analysis based on metabolome and transcriptome

We searched the differentially expressed genes (DEGs) that are involved in the flavonoid biosynthesis, which is well annotated, since the anthraquinone biosynthesis pathway is poorly annotated. Correlation analysis between compound peak intensity and DEGs expression level was conducted using the SPSS 24.0 software. According to the content difference of physcion in ROB and RPL, DEGs annotated as caffeoyl coenzyme methyltransferase were picked as candidates of key genes involved in the anthraquinone biosynthetic pathway, which were further validated by RT-qPCR.

### Quantitative reverse transcription polymerase chain reaction

All the DEGs were subjected to real-time quantitative PCR (RT-qPCR) on an ABI StepOnePlus Real-Time PCR System (Applied Biosystems, USA) for gene validation and expression analysis. Primer sequences designed using the Primer Premier 5.0 software are shown in Table S1. The primer design was based on the common PCR primer design, the optimal length of the RT-qPCR products were 80–180 bp, and the annealing temperature of the primers was 55–58 °C. Furthermore, cDNA synthesis and RT-qPCR were performed using a FastKing cDNA RT Kit (With gDNase) (TIANGEN Biotech (Beijing) Co., Ltd., China) and a TransStart Green qPCR SuperMix UDG Kit (TransGen Biotech Co., Ltd.). The template was diluted to approximately 1 μg/μL. The RT-qPCR reaction system was listed (Table S2), and the qPCR reaction program involved incubation at 95 °C for 2 min, denaturation at 94 °C for 15 s, annealing at 60 °C for 30 s, and extension at 72 °C for 30 s, for a total of 40 cycles. Actin was selected as an internal control. The relative expression of specific genes was quantified using the 2^−ΔΔCt^ calculation method^41^. Six independent biological replicates for each sample and three technical replicates of each biological replicate were arranged to ensure the reproducibility and reliability of RT-qPCR results.

## Supplementary information

Supplementary Information 1.

## Data Availability

The RNA-seq data reported in this paper have been deposited in the genome sequence archive of Beijing Institute of Genomics, Chinese Academy of Sciences. (gsa.big.ac.cn, Accession No. PRJCA002893).
