# Supplementary material for: Identification and quantification of target metabolites combined with transcriptome of two rheum species focused on anthraquinone and flavonoids biosynthesis
Source: Sci Rep. 2020 Nov 20;10:20241. doi: 10.1038/s41598-020-77356-9 (PMC7679448; doi:10.1038/s41598-020-77356-9)
Supplement: Supplementary file 1 — Supplementary Information 1. [file 41598_2020_77356_MOESM1_ESM.docx]

**Identification and Quantification of Target Metabolites Combined with Transcriptome of Two Rheum Species Focused on Anthraquinone and Flavonoids Biosynthesis**

**Jing Liu, Liang Leng, Yan Liu, Han Gao, Wei Yang, Sha Chen*, An Liu***

*Key Laboratory of Beijing for identification and safety evaluation of Chinese medicine, Institutute of Chinese Materia Medica, China Academy of Chinese Medical Sciences, No.16, Nanxiaojie, Dongzhimennei, Beijing 100700, China*

*Corresponding authors: schen@icmm.ac.cn (S. Chen) and aliu@icmm.ac.cn (A. Liu)

Figure S1. HPLC elution profiles of the metabolites from ROL leaves. MS chromatography was monitored at 280 nm.


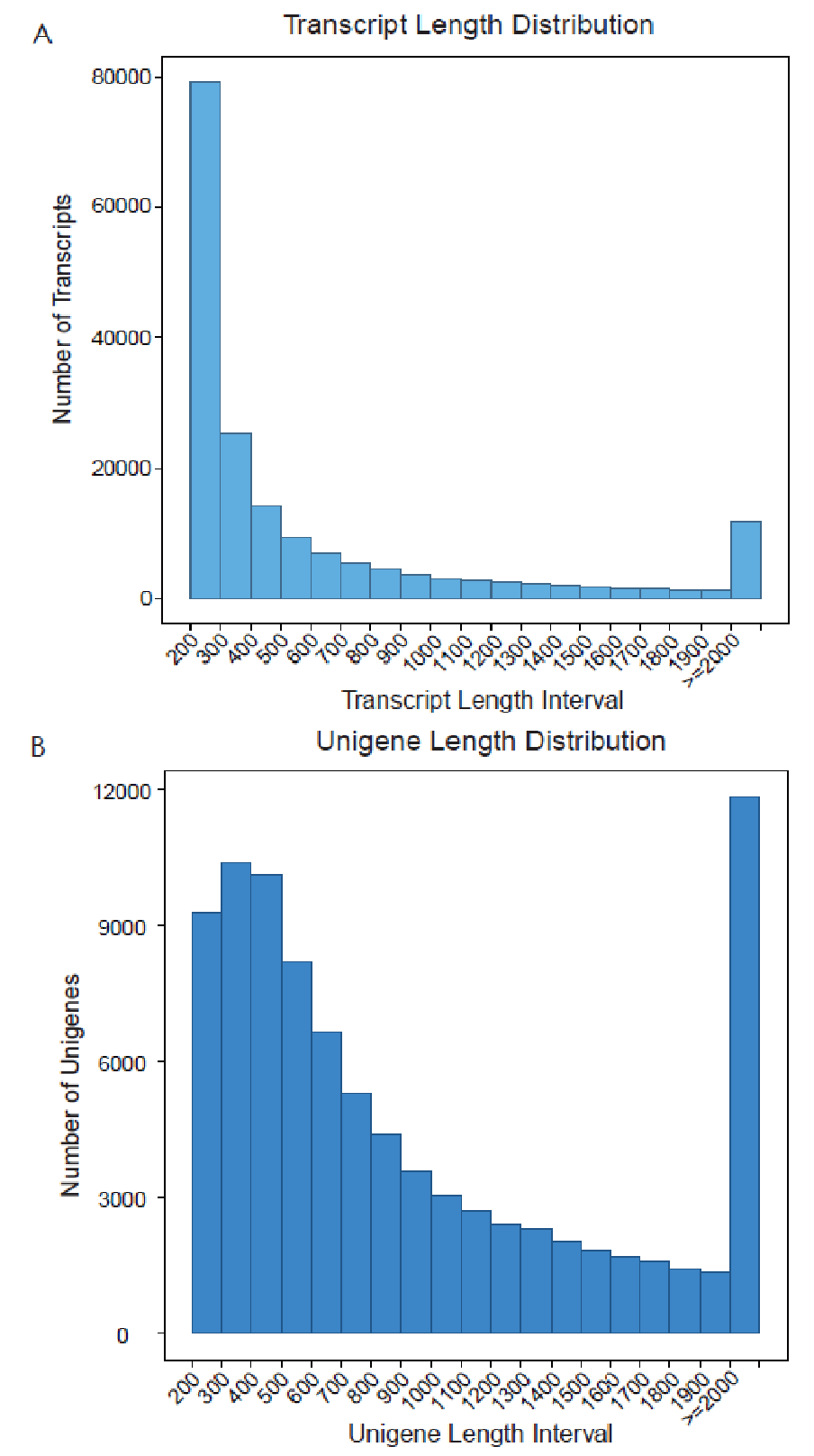


Figure S2. Overview of the de novo transcriptome assembly. (A): Length of transcripts assembled from high-quality clean reads using the Trinity program. (B): Length of unigens generated from further transcript assembly.


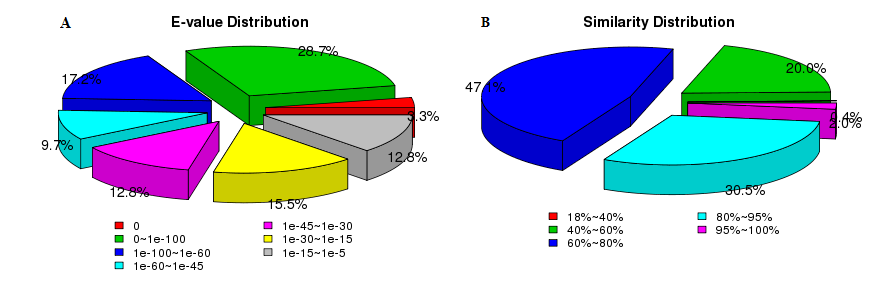


Figure S3. Characterization of the assembled unigenes based on a search performed in the Nr protein database. (A): E-value distribution of BLAST hits for the assembled unigenes, with a cutoff E-value of < 10−5. (B): Similarity score distribution of the top BLAST hits for the assembled unigenes, with a cutoff E-value of < 10−5.


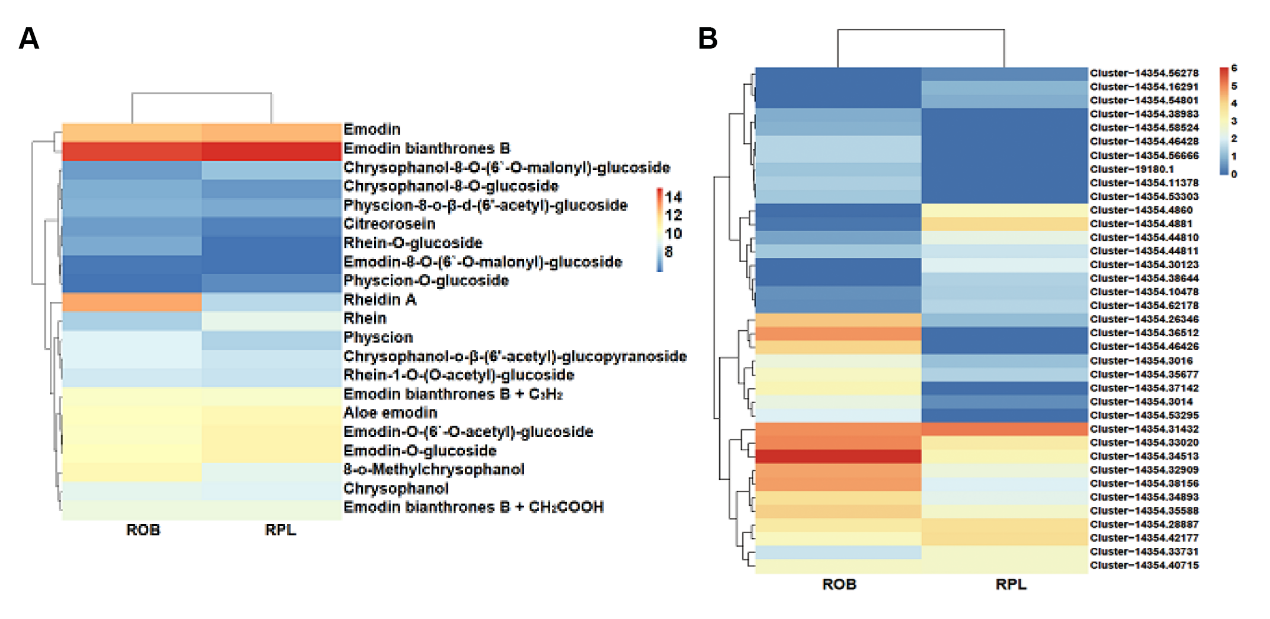


Figure S4. The heat map of the content of 21 anthraquinones in ROB and RPL and the 38 DEGs expressions annotated by flavonoid pathway. (A) The content differences of anthraquinones in two Rhubarbs; (B) The expression differences of 38 genes in flavonoids pathway.

Table S1. List of primers for RT-qPCR.

| No | Gene ID | Primer (5’-3’) |
| --- | --- | --- |
| 1 | Cluster-14354.34366 | qF-GATGGAGTGAGCCACACAGTT |
| 2 |  | qR-TCAGCAGATGTTGTCAGCGAG |
| 3 | Cluster-14354.36512 | qF-TGCCGACAAGGACAACTATCT |
| 4 |  | qR-CGTTCCATAGGGTGTTGTCGT |
| 5 | Cluster-14354.38156 | qF-TCACCGCAAACCACCCAT |
| 6 |  | qR- GGTGGCAAGGAGGGAGTA |

Table S2. Reaction system for RT-qPCR.

| Components | Volume (Total Volume 20 μl) |
| --- | --- |
| 2×TransStart Green qPCR SuperMix UDG | 10 μl |
| Templet | 1 μl |
| Forward primer (10 μM) | 0.4 μl |
| Reverse primer (10 μM) | 0.4 μl |
